# Research Progress of Polysaccharide-Based Natural Polymer Hydrogels in Water Purification

**DOI:** 10.3390/gels9030249

**Published:** 2023-03-20

**Authors:** Wenxu Zhang, Yan Xu, Xuyang Mu, Sijie Li, Xiaoming Liu, Ziqiang Lei

**Affiliations:** 1 Key Laboratory of Eco-Functional Polymer Materials of the Ministry of Education, Northwest Normal University, Lanzhou 730070, China; 2 College of Chemistry and Chemical Engineering, Northwest Normal University, Lanzhou 730070, China; 3 Key Laboratory of Eco-Environmental Polymer Materials of Gansu Province, Northwest Normal University, Lanzhou 730070, China

**Keywords:** freshwater resources, hydrogel, natural polymer materials, adsorption properties

## Abstract

The pollution and scarcity of freshwater resources are global problems that have a significant influence on human life. It is very important to remove harmful substances in the water to realize the recycling of water resources. Hydrogels have recently attracted attention due to their special three-dimensional network structure, large surface area, and pores, which show great potential for the removal of pollutants in water. In their preparation, natural polymers are one of the preferred materials because of their wide availability, low cost, and easy thermal degradation. However, when it is directly used for adsorption, its performance is unsatisfactory, so it usually needs to be modified in the preparation process. This paper reviews the modification and adsorption properties of polysaccharide-based natural polymer hydrogels, such as cellulose, chitosan, starch, and sodium alginate, and discusses the effects of their types and structures on performance and recent technological advances.

## 1. Introduction

Water is one of the most valuable resources on the earth and is closely related to the survival and development of human beings and other organisms. With the development of industrialization and modernization, water pollution is becoming more and more serious. Pollutants, such as heavy metal ions, dyes, and drugs from paint, mining, energy storage, oil, textiles, medicine, food, and printing industries, are discharged into the environment [1]. The United Nations World assessment of water resources reported that about 2 million tons of garbage was dumped into rivers, lakes, and streams every day in the world, and 3 million to 4 million people die of water-related diseases every year. It predicts that in 2025, the water crisis will spread to 48 countries, and 3.5 billion people will be trapped by water. Therefore, effective new methods must be developed, studied, and implemented to improve the pollution of water resources.

There are many means currently used for water treatment. For example, the electrochemical method [2], flocculation [3], membrane separation [4], ion exchange [5], and adsorption [6]. Electrochemical methods require fewer chemicals and no secondary pollution formation but are expensive and technically difficult. Compared with the above methods, the adsorption method has received a lot of attention due to its high removal efficiency, good economic efficiency, and flexible operation. At present, the common adsorbent materials mainly include new magnetic nano-adsorbent materials, metal-organic framework adsorbent materials, bio-carbon adsorbent materials, microbial adsorbent materials, natural polymer adsorbent materials, etc. Single adsorbent materials have more limitations in their application, but the composite of adsorbent materials can combine the advantages of several materials in one, achieving high performance and low cost of materials. Among them, natural polymer adsorbent materials with excellent biocompatibility and economic benefits are used as one of the main synthetic hydrogel adsorbent materials. In addition, hydrogels compounded with natural polymer materials generally have a large specific surface area and special micro- and nanostructure and pore structure; and contain various hydrophilic functional groups, such as R-OH, R-COOH, R-NH_2_, R-CONH_2_, R-SO_3_H, etc. The above functional groups provide multiple adsorption sites for the removal of ionic pollutants and ensure the possibility of hydrogel modification, which is preferred for the preparation of natural hydrogel adsorbent materials [7,8].

This paper presents a review of the current state of research on polysaccharide-based natural polymeric hydrogel adsorbent materials for pollutant removal from water. An overview of the modification problems in their preparation is given, and an explanation of the mechanisms related to pollutant removal is highlighted. The categories and applications of natural polymer hydrogels are so broad that it is difficult to explain them in one article. Therefore, this article concentrates on the progress of water treatment applications of polysaccharide-based hydrogels among natural polymer hydrogels and analyzes them from various aspects, such as stability, reusability, mechanical properties, and the recycling of current hydrogels.

## 2. Preparation and Modification of Polysaccharide Hydrogels

### 2.1. Cellulose-Based Hydrogels

**Figure 1:**
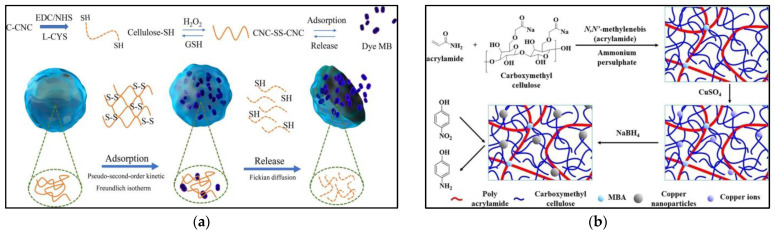
(**a**) The adsorption and release mechanisms of MB on gel-like adsorbent Cellulose-SH. [12]; (**b**) schematic shows the preparation of the CMC/PAM composite hydrogel, the adsorption of CuII ions, the formation of Cu NPs in the hydrogel network, and the catalytic reaction of 4-NP to 4-AP [13]. Informed consent was obtained from all subjects involved in the study.

Cellulose, one of the most widely distributed polysaccharides in nature, has the advantages of being hydrophilic and biodegradable, making it one of the most renewable and available polymer resources today. It can be used as an inexhaustible material in the context of the increasing demand for environmentally friendly and biocompatible products [9]. Cellulose is rich in hydroxyl groups, which can be modified by a surface modification to impart higher chemical activity, and successfully modified cellulose materials can improve adsorption properties. Grafting is one of the most used modifications. Polyethyleneimine is grafted onto oxidized cellulose hydrogels, and the addition of amine groups improves the adsorption. Grafting 2-acrylamido-2-methylpropane sulfonic acid (AMPS) onto cellulose-based hydrogels with negative sulfonic acid groups on the chain improves the adsorption efficiency of the hydrogels for metal cations or cationic dyes. In addition to traditional modifications, fluorescence technology has also been chosen to be combined with hydrogels [10]. Xia Lei et al. [11] combined fluorescence technology with nanocellulose, a new fluorescent nanocellulose hydrogel that can sensitively perform selective detection, and the adsorption of heavy metal Hg^2+^ with a maximum adsorption capacity of 95.7 mg/g was prepared, which provides the possibility of specific and selective adsorption of the hydrogel. As shown in Figure 1a, Yuchen Li et al. [12] synthesized redox-responsive hydrogels by using the reaction of activated carboxymethyl cellulose nanocrystals with L-cysteine, and the highlight of this article is its ability to use the reducing agent glutathione to achieve a controlled release of methylene blue dye with an adsorption capacity of 756 mg/g. Natural hydrogels can also be linked to catalytic applications, as shown in Figure 1b, where Chirag B. et al. [13] showed that after the in situ reduction of the hydrogel after the adsorption of Cu^2+^ ions, copper nanoparticles uniformly overlaid on the hydrogel could efficiently reduce 4-nitrophenol to 4-aminophenol in a study that enabled the realization of a hydrogel with dual functions for wastewater treatment and catalytic applications.

However, the presence of a large number of hydroxyl groups in the molecular chain, which are prone to hydrogen bonding, poses a challenge to its processing and application, limiting the further expansion of its applications. In addition, cellulose-based hydrogels still suffer from poor mechanical properties and low biocompatibility, which affect their recycling in the field of adsorption.

### 2.2. Chitosan-Based Hydrogels

**Figure 2:**
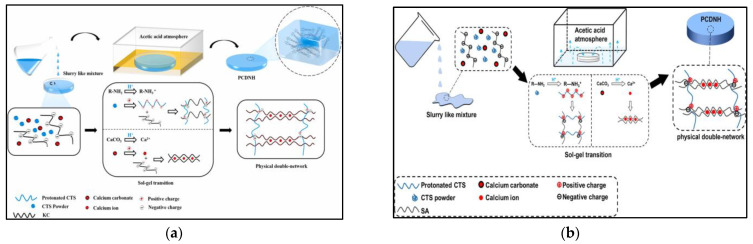
(**a**) Formation mechanism of KC/CTS/Ca^2+^ PCDNH [22]. (**b**) Schematic illustration of the preparation process of CTS/SA/Ca^2+^ PCDNH [24]. Informed consent was obtained from all subjects involved in the study.

Chitosan is a partially deacetylated product of chitin, which is widely found in shrimp and crab shells [14]. Chitosan can be divided into high molecular weight chitosan and low molecular weight chitosan, and chitosan with different molecular weights exhibits different properties. It was found that under acidic conditions, high molecular weight chitosan (HMW:120~150 kDa) had the highest adhesion, while low molecular weight chitosan (HMW: ~5 kDa) had the lowest adhesion; therefore, the suitable molecular weight chitosan can be selected according to different needs [15]. In performance, chitosan is biodegradable, regenerable, and non-toxic; the structure contains many amino and hydroxyl groups, which provide adsorption sites for the removal of dyes and heavy metal ions, enriching the mode of action in the adsorption process and ensuring the possibility of modification. Researchers have used grafting, impregnation, blending, interpenetration, and the molecular imprinting of inorganic materials to physically and chemically modify chitosan materials as a way of improving the adsorption and mechanical properties of hydrogels. Magdalena Blachnio et al. [16] prepared the hydrogel by grafting the inorganic material SiO_2_ with chitosan for the removal of sulfonated azo dyes from aqueous solutions, which had an excellent adsorption rate, removing 50% of the dye from the solution in only 5 min. Hui Deng et al. [17] molecularly imprinted chitosan’s molecular surface with Ti^4+^ as a Lewis acid. This technique can specifically adsorb the imprinted contaminants; the hydrogel prepared with this technique uses titanium’s properties to imprint, identify, and adsorb the dye reactive brilliant red X3-B with a maximum adsorption capacity of 161.1 mg/g. Lutiane N. Affonso et al. [18] used carbon nanotubes to adsorb fluorine from acidic fertilizer industry wastewater by combining the adsorption and mechanical properties of carbon nanotubes with the highly porous structure of biopolymers, which had an adsorption capacity of about 975.4 mg/g. S.-C. Yang et al. [19] prepared the cellulose-based composite hydrogel via co-melting and regeneration processes. The addition of chitosan introduced both the metal adsorption function and increased the specific surface area of the composite hydrogel to improve its mechanical strength, and the adsorption amount of Cu^2+^ reached 94.3 mg/g. In addition, chitosan can also be used with organic materials [20,21], polysaccharides [22], amino acids [23], and other modified materials compounds as shown in Figure 2a,b.

### 2.3. Starch-Based Hydrogels

**Figure 3:**
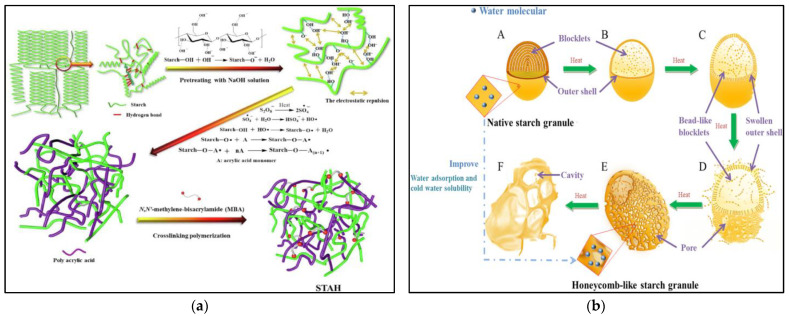
(**a**) Schematic illustration of the synthesis process and proposed chemical structure of the STAH [28]. (**b**) Schematic diagrams for the morphological changes during the formation of honeycomb-like starch granules [29]. Informed consent was obtained from all subjects involved in the study.

Starch is the second most abundant polysaccharide in nature and can be extracted from lower-cost natural products from a wide range of sources [25]. Starch can be divided into straight-chain starch and branched-chain starch. Straight-chain starch is mainly composed of linear chains of glucose units connected by α-1–4 with low molecular weight and a handful of long chain branches; in contrast, branched-chain starch is a highly branched molecule connected by α-1,4- and α-1,6-glycosidic bonds with high molecular weight and a large number of short-chain branches [26]. Natural starches lack some functional groups that are structurally necessary for water treatment and have low adsorption capacities, so natural starches must be modified before use. There are many methods of modification, such as grafting, cross-linking, pasting, esterification, oxidation, and radiation [27]. As shown in Figure 3a, Liwei Chen et al. [28] grafted polyacrylic acid onto starch to enrich the functional groups of the hydrogel with an adsorption capacity of 2967.66 mg/g. Chenlu Fang et al. [29] altered the internal structure of starch by means of pasting, as shown in Figure 3b. The high-temperature pasted starch had a honeycomb shape inside with improved dispersibility, adsorption capacity, and cold-water solubility compared to natural starch. Liping Bao et al. [30] grafted succinic acid molecules onto potato starch chains and successfully modified the starch by breaking the hydrogen bonds inherent in the starch macromolecule chains. The addition of mercaptosuccinic acid molecules introduced -COOH, which increased the adsorption sites of the gels and significantly enhanced the adsorption capacity. In addition, Qi-Jie Chen et al. [31] also used succinic anhydride to chemically modify starch nanocrystals to prepare hydrogels that can adsorb Cu^2+^ and methylene blue dyes. Li Guo et al. [32] explored a novel dual enzyme combination to modify starch, and the combination of cis-glycosyltransferase and branching enzymes that he chose could produce more large pore structures (>50 nm). Its adsorption capacity was twice as high as that of conventional enzyme modification methods. The reason is that the glycosyltransferase causes the starch to form a greater density of branched chains and more short-branched chains. The modified starch has 8–16 times higher oil absorption capacity and 6–11 times higher heavy metal adsorption capacity than the natural starch, providing a new method for the modified synthesis of starch-based composites.

### 2.4. Sodium-Based Hydrogels

**Figure 4:**
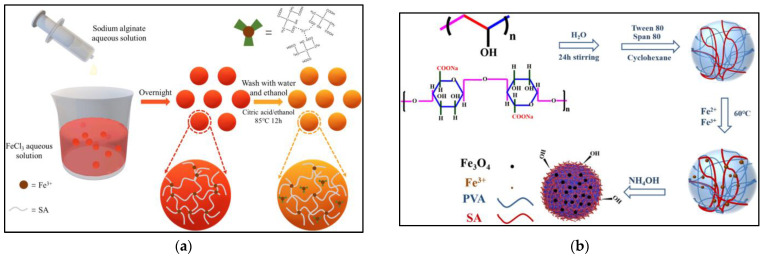
(**a**) Pathway of the preparation of hydrogel spheres [35]. (**b**) Schematic synthetic route of the alginate/PVA magnetic microspheres [38]. Informed consent was obtained from all subjects involved in the study.

Sodium alginate is one of the typical representatives of natural hydrogel materials, and its most important feature is that metal ions can be used as cross-linking agents to cross-link alginate to form spherical gel materials. It is also an anionic polymer with abundant internal hydroxyl and carboxyl groups, which can be cross-linked with divalent metal ions to form a stable three-dimensional network structure backbone. However, the low physical strength and poor thermal stability of sodium alginate greatly limit its application in water treatment; therefore, physical or chemical modifications are needed to improve its applicability in the field of adsorption [33]. Dianjia Zhao et al. [34] prepared sodium alginate beads for Cu^2+^ adsorption by cross-linking the interaction between sodium alginate solution and calcium ions. Yueshan Li et al. [35] innovatively prepared hydrogels with multilayer pore structures and photochromic abilities for the effective photocatalytic degradation of malachite green, as shown in Figure 4a. Under 365 nm UV light irradiation, about 95% of the initial concentration of 20 mg/L malachite green could be degraded in about 4 min. Tannaz Mozaffari et al. [36], by incorporating copperII tetraamine sulfate into the sodium alginate complex, found more than 95% of the adsorbed material remained available for adsorption after eight adsorption cycles. Jie Ma et al. [37] prepared triple network composite hydrogels with excellent mechanical properties by combining inorganic carbon nanotube and graphene oxide materials with sodium alginate with the help of hydrogen peroxide and L-cysteine to achieve the efficient removal of the antibiotic ciprofloxacin. In addition, as shown in Figure 4b, Man-Ke Zhang et al. [38] prepared a semi-interpenetrating network of hydrogel materials with magnetic properties and the selective adsorption of contaminants by compounding Fe_3_O_4_ with sodium alginate.

## 3. Adsorption Mechanism and Kinetics of Polysaccharide-Based Hydrogels

The adsorption of the hydrogel can be divided into chemisorption and physisorption. Chemisorption is an irreversible process, mainly because its adsorbent and adsorbent are chemically bonded in the interaction; the destruction of the bond is permanent and, once destroyed, will not be able to bond again. In contrast, physisorption is an irreversible process, usually controlled by physical forces, such as hydrogen bonding, ionic bonding, π–π stacking, hydrophobic interactions, etc., and can be restored after being disrupted. In the preparation of hydrogels, physical action is usually combined with chemical action to strengthen their adsorption properties and mechanical properties, such as in self-healing hydrogels, which usually disperse energy consumption by physical action to ensure that the integrity of internal chemical bonds and mechanical properties is improved; such hydrogels are widely used in bioengineering [39]. In the preparation of hydrogels in the field of adsorption, researchers have also chosen to combine the two modes of action. The specific adsorption mechanisms and their classification are as follows.

### 3.1. Adsorption Mechanism

**Figure 5:**
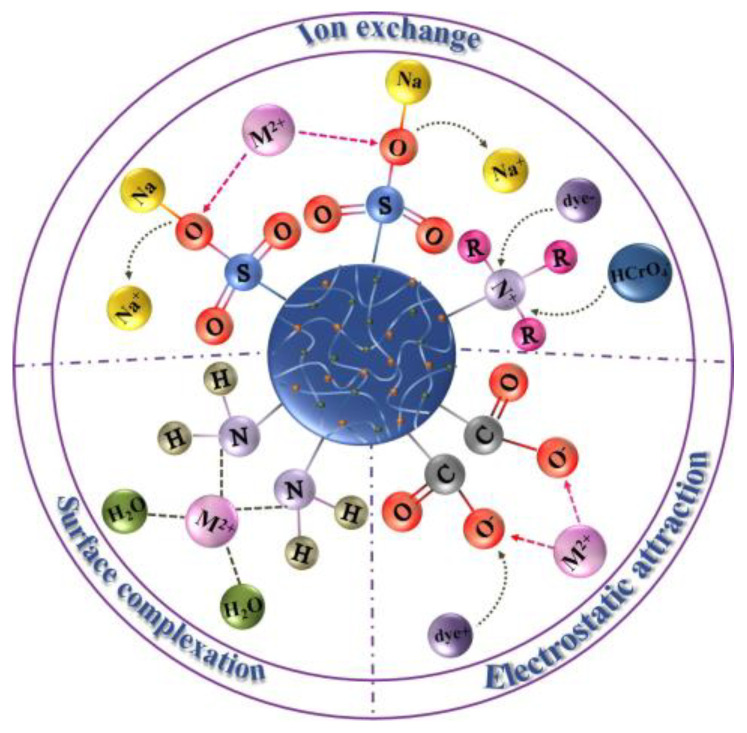
Schematic diagram of the adsorption process in three modes.

In the adsorption process, different functional groups have different adsorption mechanisms and different modes of action, and functional groups determine the type and strength of intermolecular forces and the chemical reactivity of molecules. The main functional groups involved in the adsorption process of polysaccharide-based hydrogel materials can be divided into three main categories: oxygen-containing functional groups, nitrogen-containing functional groups, and sulfur-containing functional groups. N, O, and S class heteroatoms can contribute to one or more electrons and form coordination bonds with metal ions while also undergoing ion exchange or electrostatic attraction to achieve the adsorption of a wide range of contaminants. Among the various metal adsorption mechanisms reported for hydrogels, the three methods of electrostatic interactions, ion exchange, and surface complexation (including coordination and chelation) have been found to be closely related to surface functional groups [6]. As shown in Figure 5, the process of action when adsorption is carried out by these three modes of action is depicted, among which most adsorption mechanisms of the two electrostatic interactions and ion exchange are reversible, and the adsorbents can be reused and belong to physical adsorption.

### 3.2. Adsorption Kinetics

#### 3.2.1. Pseudo-First-Order Kinetic

The pseudo-first-order kinetic model is based on the modal diffusion theory, which assumes that the arrival of the adsorbent from the solution to the adsorbent’s surface is controlled by the diffusion step and that the adsorbent’s surface has only one binding site [40]. The form of the equation is as follows:

(1) $$\ln\left(q_{e}-q_{t}\right){=\mathrm{lnq}}_{e}-K_{1}t$$

where q_t_ is the adsorption capacity at time t (mg/g), q_e_ is the adsorption capacity at the moment of adsorption equilibrium (mg/g), and K_1_ is the rate constant of the first-order kinetic.

Although the first-order kinetic model has been widely used for various adsorption processes, it has limitations. It is often only suitable for the kinetic description of the initial stage of adsorption and cannot accurately describe the entire process of adsorption [41].

#### 3.2.2. Pseudo-Second-Order Kinetic

The pseudo-second-order kinetic model is based on the adsorption rate-limiting step and contains the adsorption mechanism, such as chemisorption, which involves electron sharing or electron transfers between the adsorbate and the adsorbent [42]. The conformity to the pseudo-second-order kinetic model indicates that adsorption kinetics are mainly controlled by chemical interactions rather than by the material transport steps. The form of the equation is as follows:

(2) $$\frac{t}{q_{t}}=\frac{1}{K_{2}q_{e}{}^{2}}+\frac{t}{q_{e}}$$

where q_t_ is the adsorption capacity at time t (mg/g), q_e_ is the adsorption capacity at the moment of adsorption equilibrium (mg/g), and K_2_ (g/mg·h) is the pseudo-second-order rate constant.

### 3.3. Adsorption Isotherms

#### 3.3.1. Langmuir Isotherm Equation

The Langmuir adsorption isotherm model is the most widely used molecular adsorption model, which can predict the maximum adsorption capacity of adsorbents by considering the influence of the adsorbent’s surface and temperature [43]. This theory is a single molecular layer adsorption theory, which requires a homogeneous solid surface with the same adsorption capacity and no interaction between the adsorbed molecules, but the assumptions of the model are far from the actual conditions, and the information obtained is sometimes highly inaccurate [44]. The form of the equation is as follows:

(3) $$\frac{C_{e}}{q_{e}}=\frac{C_{e}}{q_{m}}+\frac{1}{q_{m}K_{L}}$$

where C_e_ is the equilibrium concentration of the solution, mg/L, q_m_ is the maximum adsorption capacity (saturation), mg/g, and K_L_ is the Langmuir constant related to the affinity and adsorption energy of the bonding site, L/g.

#### 3.3.2. Freundlich Isotherm Equation

The Freundlich isothermal adsorption equation is an empirical equation with no assumptions. The form of the equation is as follows:

(4) $${\mathrm{lnq}}_{e}{=\mathrm{lnK}}_{F}+\frac{1}{n}{\mathrm{lnC}}_{e}$$

where q_e_ is the adsorption amount, mg/g, when adsorption reaches equilibrium, C_e_ is the concentration of adsorbate in solution at adsorption equilibrium, mg/L, K_F_ is the constant related to adsorption capacity and adsorption strength under the Freundlich model, and 1/n is the Freundlich constant. A large value of K_F_ is a sign of a better adsorption performance of the adsorbent. Freundlich adsorption isotherms can be obtained by plotting lnq_e_ against lnC_e_ at different temperatures [45].

## 4. Modification of Polysaccharide-Based Hydrogels

### 4.1. Functionalization of Nitrogen-Containing Groups

The nitrogen-containing functional groups mainly include an amine group (R-NH_2_), an amide group (R-CONH_2_), a quaternary amine group (R-NH_4_^+^), and so on. Amine groups contain a lone pair of SP_3_-hybridized electrons, which can be coordinated with empty orbitals and combined with contaminants. They have good protonation ability under acidic conditions and can adsorb positively charged contaminants by electrostatic attraction and ion exchange. In addition, the amine group is also easily functionalized, and the characteristic reaction of the amine group can be used to enrich the functional groups on the main chain and increase the adsorption performance. Nitrogen-containing compounds can be introduced into polysaccharide-based hydrogels by grafting amines on the surface of materials such as polyethyleneimine (PEI), ethylenediamine (EDA), and p-phenylenediamine (PDA) [46,47,48]. Chitosan-polyethyleneimine hydrogels were prepared using calcium chloride as an ionic cross-linking agent. The addition of polyethyleneimine increased the number of amino groups in the adsorbent, leading to an increase in the adsorption capacity of AR88 dye up to 1000 mg/L. In addition, the modified adsorbent had a higher specific surface area, porosity, and thermal stability compared to the unmodified adsorbent [49]. The nucleophilic addition reaction of ketones and amine groups can also be used to modify chitosan by grafting the carboxyl group in α-ketoglutaric acid onto chitosan, which increases the active site for the adsorption of heavy metal ions and improves the adsorption performance of the hydrogel [50]. Compared to amine groups, the role of amide groups is not significant in the field of hydrogel adsorption. Amide groups are bonds between nitrogen atoms and carbonyl groups, and common amide-based monomers include polyacrylamide, 2-acrylamido-2-methyl-1-propanesulfonic acid (AMPS), etc. When AMPS is used for adsorption, in addition to amide groups, negatively charged sulfonic acid groups (R-SO_3_^−^) also play a major role [51]. Quaternary ammonium salt (R-N^+^(CH_3_)^3^) compounds are the most active and have a powerful affinity for R-CrO_4_^2−^, R-HCrO_4_^−^, R-Cr_2_O_7_^2−^, and R-AsO_4_^3−^, which contain negative metal oxygen ions. Saltuk Pirgalıog˘lu et al. [52] used diallyldimethylammonium chloride (DADMAC) and N,N′-tetraallylpiperaziniumdi-chloride (TAP) that were cross-linked and co-polymerized into high-porosity cationic hydrogels with high affinity over a wide pH range for arsenate anions. Hemant Mittal et al. [53] also used DADMAC as a monomer to synthesize hydrogels that can adsorb both anionic and cationic dyes.

### 4.2. Functionalization of Oxygen-Containing Groups

The surface modification of hydrogels by the “O” comprising functional groups, specifically –OH, and –COOH is considered to enhance the adsorption efficiency of water pollutants. The chemical structure of the carboxyl group contains carbonyl (-C=O) and -OH groups, which lose their hydrogen ions to carboxylic acid negative ions (R-COO^−^), and the negatively charged carboxylic acid ions electrostatically attract oppositely charged divalent metal cations and cationic dyes [10]. In addition to this electrostatic interaction, carboxyl groups can also be functionalized to ligate with metal ions for adsorption [54]. Liping Bao et al. [30] introduced a carboxylic acid group (R-COOH) into the hydrogel, and the adsorption power was substantially increased compared to the unmodified gel. For the hydroxyl group, it is easily deprotonated to form its conjugate base, which attracts positively charged metal cations and cationic dyes. In addition, the hydroxyl group can also undergo ion exchange with metal ions for the purpose of adsorption. Jinsong He et al. [55] prepared a composite hydrogel of sodium alginate and graphene oxide, which used the hydroxyl group in sodium alginate to adsorb As^5+^ in water by ion exchange with H_2_AsO_4_^2−^.

### 4.3. Functionalization of Sulfur-Containing Groups

The functional groups containing sulfur include thiols (R-SH), sulfonic acid groups (R-SO_3_H), etc. When a sulfur atom replaces an oxygen atom in the -OH of alcohol, a thiol group is formed, and when thiols are present on the surface of hydrogels, they interact mainly with metal ions since thiols act as Lewis bases and bind to metals through coordination bonds; this group is quite abundant in protein structures such as glutathione and cysteine. Haiwang Lai et al. [56] prepared hydrogels by alternating the copolymerization of L-cysteine and itaconic anhydride, which exhibited strong silver ion-trapping abilities. The chemical structure of the sulfonic acid group (R-SO_3_H) contains a negatively charged sulfur atom that is double bonded to two oxygen atoms and single bonded to the R-OH group, and the hydrogen ion of the sulfonic acid group dissociates into the starting conjugate base (R-SO_3_^−^), which makes the surface of the hydrogel negatively charged and able to adsorb metal ions. We use 2-acrylamido-2-methylpropane sulfonic acid (AMPS) as an example. Ahmed Mohamed Omer et al. [57] made semi-interpenetrating hydrogels with polyvinyl alcohol and poly-2-acrylamido-2-methylpropane sulfonic acid (PAMPS) for the adsorptive removal of cationic methylene blue (MB) dyes. Marzieh Aflaki Jalali et al. [58] cross-linked and copolymerized xanthan gum with AMPS and prepared hydrogels with side chains rich in hydroxyl groups, carboxyl groups, and sulfonates for the effective removal of Cu^2+^.

## 5. Adsorption Applications of Polysaccharide-Based Hydrogels

### 5.1. Heavy Metal Ion Adsorption

Heavy metal ions are highly toxic, non-degradable, and bioaccumulative in the environment and circulate through the food chain in water and biological systems, seriously affecting the organisms at the top of the food chain [59,60]. Heavy metals are metals with a density greater than 4.5 g/cm^3^, mainly including Au, Ag, Cu, Pb, Zn, Ni, Co, Cd, Hg, Cd, and more than 40 other kinds of metals [61]. The five most toxic to humans are lead, mercury, chromium, arsenic, and cadmium. These heavy metals cannot be decomposed in water, and their toxicity is amplified when they enter the human body; therefore, efficient and special methods are needed to remove heavy metal contaminants from water systems [62]. The adsorption process of heavy metal ions is directly related to the functional groups of the adsorbent materials themselves, and most current studies mainly revolve around the adsorption of divalent heavy metal ions, of which Cu^2+^, Pd^2+^, and Cd^2+^ are predominant. Table 1 summarizes the polysaccharide-based adsorbent materials in the last two years for this application of heavy metal ion adsorption.

### 5.2. Dye Adsorption

There are many methods for removing dyes from wastewater: biological dye removal, acoustic chemical degradation, electrocatalytic degradation, cation exchange membrane technology, etc. However, these processes produce toxic residues that cause secondary pollution and are costly to implement. In contrast, the gel’s adsorption method is simple, efficient, and inexpensive to operate. Hydrogels have a strong dye removal capability, and dyes are more easily diffused in the dissolved hydrogel, which enhances the adsorption capacity via electrostatic interactions with oppositely charged dyes [85].

There is a wide range of adsorbed dyes, among which methylene blue (MB), malachite green (MG), and methyl orange (MO) fuels are more widely adsorbed. MB is a phenothiazine cationic dye, an alkali, that is used to treat methemoglobinemia in histology and microscopy to identify and detect bacteria, to treat fungal infections by staining tissues [21], and to stain cotton and wood. The waste dye discharged into the environment after use can be harmful, causing dizziness, headaches, tremors, and mental confusion, among other symptoms [86]. Malachite green (MG) is a toxic trityl methane chemical, both as a dye and as a bactericidal and parasiticidal chemical, is an alkali, and is prohibited for use in aquaculture. In industries, it is used to color leather, paper, cotton, and silk. However, it is potentially carcinogenic, teratogenic, and mutagenic and is difficult to remove from water [87]. Table 2 summarizes polysaccharide-based adsorbent materials used in the past two years for this application of dye adsorption.

### 5.3. Drug Antibiotics Adsorption

In the treatment of contaminants in water, attention has been focused mainly on the adsorption of textile dyes and heavy metals. However, the harmful effects of these emerging contaminants, such as pesticides, herbicides, fungicides, pharmaceutical compounds, and personal care products, on the water environment cannot be ignored. Once the toxic substances of pharmaceutical and medical waste enter the soil, they will be adsorbed by the soil, pollute the soil, kill microorganisms and protozoa in the soil, and destroy the microecology in the soil, which in turn will reduce the soil’s ability to degrade pollutants. Furthermore, the acid, alkali, and salts in the substances will change the nature and structure of the soil, leading to the acidification, alkalization, and hardening of the soil, affecting the development and growth of plant roots, and damaging the ecological environment; meanwhile, many harmful drug pollutants can cause serious damage to the liver and nervous systems. Table 3 summarizes the polysaccharide-based adsorption materials used for antibiotic adsorption in the past two years.

## 6. Perspectives and Recommendations

This paper briefly and systematically describes the applications and recent progress of several polysaccharide-based natural polymer gels, namely cellulose, chitosan, starch, and sodium alginate, for water treatment, focusing on the modification of materials and the adsorption mechanisms. They show excellent adsorption and separation properties for various aqueous pollutants, but low cycle times and weak mechanical strength are the main problems for their commercialization in industrial wastewater treatment; conventional hydrogels tend to weaken and lose their mechanical strength upon repeated swelling. Therefore, it is important to improve the mechanical durability of hydrogels while increasing the self-healing ability after swelling states. These issues still require extensive reliability testing. This review article provides relevant information for the design of new hydrogels with the desired functionality that can inform the refinement of the above challenges and drawbacks.

## Figures and Tables

**Table 1:** Adsorption of heavy metal ions by different polysaccharide-based composite hydrogels.

Polysaccharide Hydrogel Adsorption	Adsorbates	Adsorption Capacity (mg/g)	Adsorption Isotherm	Adsorption Kinetics	Ref.
AM/AA	Cu^2+^	157.51	Langmuir	PSO	[63]
	Pd^2+^	393.28	Langmuir	PSO	
	Cd^2+^	289.97	Langmuir	PSO	
Nanocellulose/Carbon dot	Cr^6+^	599.9	Freundlich	PSO	[64]
Straw cellulose	Cd^2+^	95.62	Langmuir	PSO	[65]
Nanocellulose/SA	Pb^2+^	318.47	Langmuir	PSO	[66]
GO-PVA-CS	Cd^2+^	172.11	Langmuir	PSO	[67]
	Ni^2+^	70.37	Langmuir	PSO	
CYCS/CNC	Pb^2+^	334.92	Langmuir	PSO	[68]
Chitosan oligosaccharide	Cr^6+^	148.1	Langmuir	PSO	[69]
α-ketoglutaric acid–PAM-CS	Cu^2+^	72.39	Langmuir	PSO	[50]
	Pd^2+^	61.41	Langmuir	PSO	
	Zn^2+^	51.89	Langmuir	PSO	
CPCS-PAM-PVA	Cr^6+^	95.31	Langmuir	PFO	[70]
Millettia speciosa Champ cellulose-CS	Cu^2+^	23.37	Freundlich	PFO	[71]
All-lignocellulose	Cu^2+^	350	Langmuir	PSO	[72]
Caffeic acid starch	Cr^6+^	96.45	Langmuir	PSO	[73]
Starch-FMBO	As^3+^	161.29	Langmuir	PSO	[74]
Starch nanoparticle	Pb^2+^	40.52	Langmuir	PSO	[27]
	Cu^2+^	32.88	Langmuir	PSO	
dibenzo-18-crown-6 starch	Cd^2+^	368.5	Freundlich	PSO	[75]
	Ni^2+^	182.5	Freundlich	PSO	
	Zn^2+^	377.5	Freundlich	PSO	
	Cu^2+^	385	Freundlich	PSO	
Succinic anhydride-SNCs	Cu^2+^	84.07	Freundlich	PSO	[31]
PVA-SA	Pb^2+^	784.97	Langmuir	PSO	[76]
ZIF-67-SA	Cu^2+^	153.63	Langmuir	PSO	[77]
AM-GO-SA	Cu^2+^	68.76	Langmuir	PSO	[78]
	Pb^2+^	240.69	Langmuir	PSO	
Zeolite-PVA-SA	Pb^2+^	99.5	Langmuir	PFO	[79]
	Cd^2+^	99.2	Langmuir	PFO	
	Sr^2+^	98.8	Langmuir	PFO	
	Cu^2+^	97.2	Langmuir	PFO	
	Zn^2+^	95.6	Langmuir	PFO	
	Ni^2+^	93.1	Langmuir	PFO	
	Mn^2+^	92.4	Langmuir	PFO	
Starch ether-SA	Cu^2+^	25.81	Langmuir	PSO	[80]
Reptilite-Starch	Pb^2+^	180.8	Langmuir	PSO	[81]
NCDs-CNF/CS	Cu^2+^	148.3	Langmuir	PSO	[82]
	Cr^6+^	294.46	Langmuir	PSO	
CTS/CA/BT	Pb^2+^	434.89	Freundlich	PSO	[83]
	Cu^2+^	115.30	Freundlich	PSO	
	Cd^2+^	102.38	Freundlich	PSO	
GO-SA	As^5+^	277.39	Langmuir	PSO	[55]
PAN-PPY-SA-GO	Cu^2+^	133.7	Redlich–Peterson	PFO	[84]
	Cr^6+^	87.2	Redlich–Peterson	PFO	

**Table 2:** Adsorption of dyes by different polysaccharide-based composite hydrogels.

Polysaccharide Hydrogels Adsorption	Adsorbates	Adsorption Capacity (mg/g)	Adsorption Isotherm	Adsorption Kinetics	Ref.
C/SA/Fe	MB	105.93	Langmuir	PSO	[88]
Carboxymethylcellulose	MB	756	Freundlich	PSO	[12]
Pineapple peel cellulose/diatomite	MB	101.94	Langmuir	PSO	[89]
PCMC-PVA	MB	172.14	Langmuir	PSO	[90]
All-lignocellulose	MB	145	Langmuir	PSO	[72]
Millettia speciosa Champ cellulose-CS	CR	221.43	Freundlich	PSO	[71]
PAM-Fe_3_O_4_-CS	MB	1603	Langmuir	PFO	[91]
Montmorillonite-CS	MB	530	Langmuir	PSO	[92]
GO-CS-Fe_3_O_4_	MB	289	Langmuir	PSO	[93]
	EBT	292	Langmuir	PSO	[94]
Jute cellulose nanocrystal	MB	131.58	Langmuir	PSO	
Succinic anhydride-SNCs	MB	84.00	Freundlich	PSO	[31]
Reptilite-Starch	MB	277.0	Langmuir	PSO	[81]
PAM-cassava starch	MB	2000	Langmuir	PSO	[95]
MXene-SA	MB	92.17	Langmuir	PSO	[96]
AA-GO-SA	MG	628.93	Langmuir	PSO	[97]
Flax seed ash-SA	MB	333.3	Langmuir	PSO	[98]
PEI-SA	MB	400	Langmuir	PSO	[47]
AM-HEMA-Starch	MG	164	Langmuir	PFO	[71]
	MV	156	Freundlich	PFO	

**Table 3:** Adsorption of drug antibiotics by different polysaccharide-based composite hydrogels.

Polysaccharide Hydrogels Adsorption	Adsorbates	Adsorption Capacity (mg/g)	Adsorption Isotherm	Adsorption Kinetics	Ref.
Fe_3_O_4_-Starch	Naphthalene	24.752	Langmuir	PSO	[99]
CS-Chitosan film	Cefotaxime Sodium	1003.64	Freundlich	PSO	[100]
GO-SA	Tetracycline	477.9	Freundlich	PSO	[55]
Amino/GO-SA	Ciprofloxacin	301.36	Langmuir	PSO	[101]
Humicacid-CS-Biochar	Ciprofloxacin	154.89	Langmuir	PSO	[102]
Biochar-CS	Ciprofloxacin	106.038	Langmuir	PSO	[103]
	Enrofloxacin	100.433	Langmuir	PSO	
GO-SA	Fluoxacin	4.11	Langmuir	PSO	[104]
	Moxifloxacin	3.43	Langmuir	PSO	
Fe_3_O_4_-SA	Tetracycline	454.54	Langmuir	PSO	[105]
	Amoxicillin	400	Langmuir	PSO	
Trimethylammonium chloride-CS	Tetracycline	22.42	Langmuir	PFO	[106]
PVA-SA-Cu^2+^	Tetracycline	231.431	Langmuir	PSO	[107]

## Data Availability

The data presented in this study are available on request from the corresponding author.

## Nomenclature

Abbreviation	Full Name
AM	Acrylamide
AA	Acrylic acid
GO	Graphene oxide
PVA	Polyvinyl alcohol
SA	Salicylic acid
CS	Salicylic acid
PEI	Polyethyleneimine ethoxylated
PAM	Polyacrylamide
CNF	Cellulose nanofibril
HPCS	Hydroxypropyl chitosan
MB	Methylene blue
MG	Malachite green
MV	Methylrosanilinium chloride
EBT	Eriochrome black T
CR	Congo red
